# Chronic kidney disease induces a systemic microangiopathy, tissue hypoxia and dysfunctional angiogenesis

**DOI:** 10.1038/s41598-018-23663-1

**Published:** 2018-03-28

**Authors:** Hans-Ulrich Prommer, Johannes Maurer, Karoline von Websky, Christian Freise, Kerstin Sommer, Hamoud Nasser, Rudi Samapati, Bettina Reglin, Pedro Guimarães, Axel Radlach Pries, Uwe Querfeld

**Affiliations:** 1 Department of Physiology, Charité Universitätsmedizin Berlin, Berlin, Germany; 2 Center for Cardiovascular Research, Charité Universitätsmedizin Berlin, Berlin, Germany; 3 Institute of Nutritional Science, University of Potsdam, Nuthetal, Potsdam, Germany; 4 Dipartimento di Ingegneria dell’Informazione, University of Padova, Padova, Italy; 5 Department of Pediatric Nephrology, Charité Universitätsmedizin Berlin, Berlin, Germany

## Abstract

Chronic kidney disease (CKD) is associated with excessive mortality from cardiovascular disease (CVD). Endothelial dysfunction, an early manifestation of CVD, is consistently observed in CKD patients and might be linked to structural defects of the microcirculation including microvascular rarefaction. However, patterns of microvascular rarefaction in CKD and their relation to functional deficits in perfusion and oxygen delivery are currently unknown. In this *in*-*vivo* microscopy study of the cremaster muscle microcirculation in BALB/c mice with moderate to severe uremia, we show in two experimental models (adenine feeding or subtotal nephrectomy), that serum urea levels associate incrementally with a distinct microangiopathy. Structural changes were characterized by a heterogeneous pattern of focal microvascular rarefaction with loss of coherent microvascular networks resulting in large avascular areas. Corresponding microvascular dysfunction was evident by significantly diminished blood flow velocity, vascular tone, and oxygen uptake. Microvascular rarefaction in the cremaster muscle paralleled rarefaction in the myocardium, which was accompanied by a decrease in transcription levels not only of the transcriptional regulator HIF-1α, but also of its target genes Angpt-2, TIE-1 and TIE-2, Flkt-1 and MMP-9, indicating an impaired hypoxia-driven angiogenesis. Thus, experimental uremia in mice associates with systemic microvascular disease with rarefaction, tissue hypoxia and dysfunctional angiogenesis.

## Introduction

Chronic kidney disease (CKD) is associated with type 4 cardiorenal syndrome and an excessive mortality from cardiovascular disease (CVD). Uremia-specific mechanisms explaining the link between CKD and CVD are incompletely understood. Accumulating evidence indicates that the earliest manifestations of CVD occur at the level of the microcirculation^1^.

The microcirculation consists of networks of arterioles, capillaries, and venules with diameter less than 300 μm^2^. It provides a vast endothelial interface between the convective blood flow system and the tissue for efficient solute and gas exchange and is actively involved in vital functions of the cardiovascular system^3^. Permanent loss of endothelial homeostasis results in target organ damage^4,5^.

Endothelial dysfunction is a sensitive and independent predictor of future cardiovascular events^6,7^ and has been consistently observed in patients with CKD^8–10^. Endothelial dysfunction might be linked to structural defects of the microcirculation including microvascular rarefaction^11^. However, it is unknown whether any CKD-induced rarefaction is sufficient to cause functional deficits in tissue perfusion and oxygen delivery. We hypothesized that microvascular disease is an early systemic process driven by the severity of CKD, preceding changes in conduit arteries and resulting in tissue hypoxia.

We here show the profound impact of CKD on architecture and function of the microvascular network *in*-*vivo*.

## Results

### Two experimental procedures generate CKD/uremia of different severity

**Figure 1:**
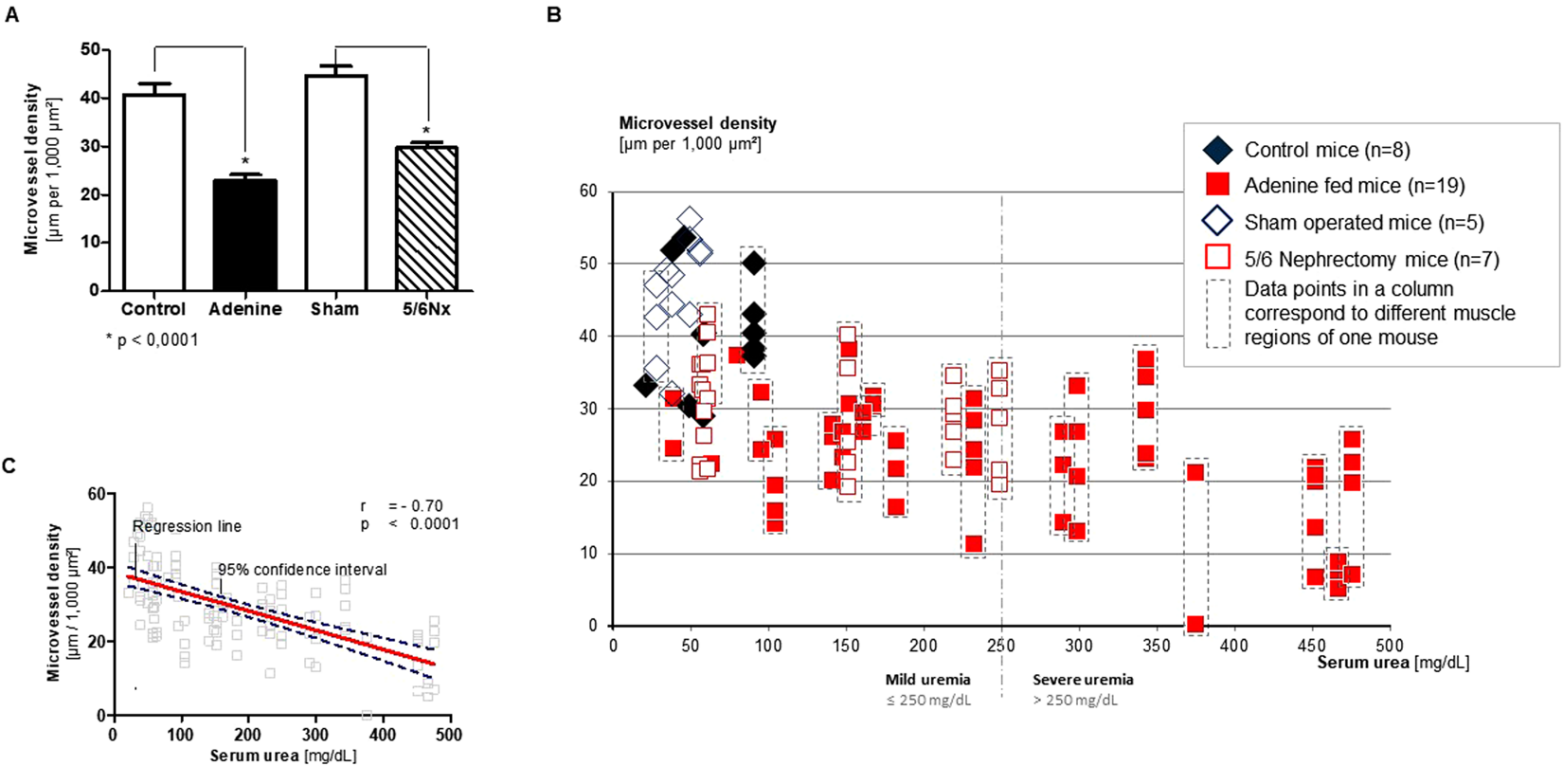
Microvascular density in the cremaster muscle correlates with the severity of uremia. Six-week-old BALB/c mice underwent a four-week adenine diet (n = 19) or 5/6 nephrectomy followed by a four-month uremia-inducing period (n = 7). Microvascular density was defined as accumulated length of microvessels [μm] per cremaster muscle area [μm²]. (**A**) For both uremia-inducing methods, mean microvascular density declines significantly (p < 0.0001, Mann-Whitney test) compared to controls (n = 8), or sham operated mice (n = 5). (**B**) Microvascular density measured as total vessel length per muscle area [µm per 1,000 µm^2^] in all experimental animals. Each column represents one studied animal, and each data point represents the microvascular density of about 800,000 µm^2^ of muscle area. (**C**) Serum urea and mean microvessel density are closely correlated (r = −0.68; p = < 0.0001, Spearman’s rank correlation coefficient).

CKD was established in mice by adenine feeding or subtotal nephrectomy. A four week 0.2% adenine diet led to serum urea levels of 39 to 475 mg/dL (median 169 mg/dL), and subtotal nephrectomy followed by a four-month uremia-inducing period led to urea levels of 57 to 249 mg/dL (median 62 mg/dL). For control and sham operated mice, the serum urea level was in the range of 21 to 91 mg/dL and 28 to 56 mg/dL, respectively. The mean urea level of uremic mice was 250 mg/dl; we arbitrarily defined a mildly uremic range (up to 250 mg/dL) and a severely uremic range (250 to 475 mg/dL) (Fig. 1). Applying those uremic ranges, we assigned animals to five groups: for the adenine model (1) controls (n = 8), (2) mildly uremic mice (n = 12), and (3) severely uremic mice (n = 7), and for the nephrectomy model (4) sham operated mice (n = 5), and (5) mice with subtotal nephrectomy (SNX; n = 7). The serum creatinine levels for these groups are shown in Supplemental Fig. 1.

Mean arterial blood pressure (MAP) measured at the day of intravital microscopy (Supplemental Fig. 2) was significantly higher in severely uremic mice compared to controls, but not significantly different between mildly uremic animals of the adenine group and their controls (p = 0.070), and SNX animals and their sham operated controls (p = 0.15) (Table 1).

**Table 1:** Characteristics of experimental groups.

	Serum urea	Body weight [g]			Mean arterial pressure [mmHg]			Hct [%]	IL-6 [pg/m]
	At intravital microscopy (IM) day	Body weight at project start	Body weight at IM day	Δ	MAP at IM start	MAP at IM end	Δ		
***Adenine induced uremia***:									
**Control** (n = 8)	**52** ± 20 mg/dl	**22**.**4** ± 0.9 g	**23**.**9** ± 0.5 g	+9%	**73**.**1** ± 2.8 mmHg	**62**.**9** ± 5.9 mmHg	−14%	**44**.**0**% ± 4.6%	**516** ± 232 pg/ml
**Mild uremia** (n = 12)	**103** ± 55 mg/dl	**21**.**9** ± 3.4 g	**21**.**4** ± 4.4 g	−2%	**76**.**0** ± 2.6 mmHg	**64**.**7** ± 2.2 mmHg	−15%	**44**.**2**% ± 5.0%	**900**^3)^ ± 251 pg/ml
**Severe uremia** (n = 7)	**386** ± 79 mg/dl	**23**.**6** ± 1.2 g	**19**.**1** ± 1.8 g	−19%	**79**.**1**^1)^ ± 2.7 mmHg	**68**.**7** ± 3.9 mmHg	−13%	**35**.**5%**^2)^ ± 7.0%	**1**,**199**^4)^ ± 261 pg/ml
**5/6 nephrectomy induced uremia**:									
**Sham** (n = 5)	**41** ± 11mg/dl	**22**.**3** ± 1.4 g	**27**.**0** ± 1.5 g	+21%	**72**.**8** ± 2.3 mmHg	**63**.**7** ± 2.7 mmHg	−13%	**43**.**8%** ± 3.0%	**593** ± 662 pg/ml
**5/6 Nx** (n = 7)	**122** ± 84 mg/dl	**23**.**2** ± 0.7 g	**26**.**5** ± 2.2 g	+14%	**77**.**0** ± 4.8 mmHg	**62**.**2** ± 4.4 mmHg	−13%	**42**.**9%** ± 4.9%	**767** ± 595 pg/ml

^1)^p = 0.005 compared to controls.

^2)^p = 0.015 compared to controls.

^3)^p = 0.032 compared to controls.

^4)^p = 0.005 compared to controls.

Interleukin-6 (IL-6), a marker of inflammation, was significantly elevated in adenine-fed mice with mild and severe uremia compared to controls (Table 1). IL-6 levels correlated significantly with urea levels (r = 0.45; p = 0.012) (Supplementary Table 1).

The hematocrit was negatively associated with serum urea (r = −0.42; p = 0.008), but not significantly different between uremic groups vs. their respective controls (Table 1).

Weight loss was observed in adenine-induced uremia (Table 1). The serum urea levels of all animals correlated with relative weight change (r = 0.33; p = 0.039); if analyzed separately in experimental groups, the correlation was confirmed in both, adenine-fed mice (r = 0.72; p = 0.0006) and SNX mice (r = 0.94; p < 0.0001). IL-6 levels were significantly associated with relative weight change (r = 0.41; p = 0.030; Supplementary Table 1).

### CKD promotes microvascular rarefaction in a dose-dependent manner

In the adenine model, the mean microvascular density of controls (n = 8), expressed as accumulated length of microvessels per 1,000 µm^2^ of cremaster muscle area was 40.7 ± 8.0 µm/kµm^2^ (Fig. 1A). The mean microvascular density of adenine-fed uremic mice (n = 19) was 23.0 ± 8.4 µm/kµm^2^, corresponding to a mean reduction of 43%. Mean microvascular density was 25.9 ± 6.3 µm/1.000 µm^2^ (−36%, p < 0.0001) in mice with mild uremia and 19.9 ± 9.3 µm/1.000 µm^2^ (51%; p < 0,0001) and severe uremia, respectively. For the nephrectomy model, the mean microvascular density was 44.7 ± 8.9 µm/kµm^2^ in sham operated mice (n = 5), and 29.7 ± 6.8 µm/kµm^2^ in SNX mice (n = 7), a mean reduction of 34%.

Microvascular density was inversely associated with the degree of experimental uremia as indicated by the serum urea level (r = −0.68; p < 0.0001; Fig. 1B and C). This association was observed in both models (r = −0.60 p < 0.0001 in the adenine and r = −0.37; p < 0.01 in the 5/6 NX model). For each 100 mg/dL increase of serum urea, a similar mean reduction of about 15% could be observed with both experimental models (14.2% for the adenine model, and 15.7% for the nephrectomy model). There was no significant correlation with the serum creatinine level in the adenine model or the SNX model.

Mean microvascular density was significantly correlated with IL-6 serum levels in the adenine model (r = −0.63; p = 0.0073), but not in the SNX model (r = −0.39; p = 0.23), and similarly, with relative weight change in the adenine model (r = 0.67; p = 0.0001), but not in the SNX model (r = 0.41;p = 0.18). Furthermore, mean vascular density was significantly associated with mean arterial blood pressure in the adenine fed mice (r = −0.54; p = 0.0034), but not in the SNX mice (p = 0.59). The correlation of microvascular density with these variables in both models combined is shown in Supplementary Table 1. In a multivariate linear regression model including all observations, only the degree of uremia as indicated by serum urea levels was an independent significant predictor of microvascular density (p < 0.0001), whereas all other variables correlating with density in univariate regression, i.e. hematocrit (p = 0.990), weight change (p = 0.220), IL-6 (p = 0.157) and MAP (p = 0.245), were excluded from the model (corrected r^2^ = 0.502).

### CKD induces a heterogeneous rarefaction pattern

Overall, microvascular rarefaction appeared highly heterogeneous in CKD animals (Fig. 1B). Some cremaster muscle areas revealed dramatic rarefaction up to complete vessel extinction, while almost no rarefaction was visible in some adjacent areas. As an example, one mouse with a serum urea level of 375 mg/dL had a cremaster observation area (800,000 μm^2^) with an almost physiological microvascular density of 21 µm/1,000 µm^2^. Yet, in a neighboring area (distance 2.02 mm), hardly any microvessel could be identified. However, since some degree of heterogeneity in microvascular density was also observed in control and sham operated mice (Fig. 1B), we analyzed the features of the CKD-induced rarefaction pattern by studying average segment lengths in all cremaster areas.

### Loss of small coherent vessel systems is the predominant mechanism or rarefaction in CKD

**Figure 2:**
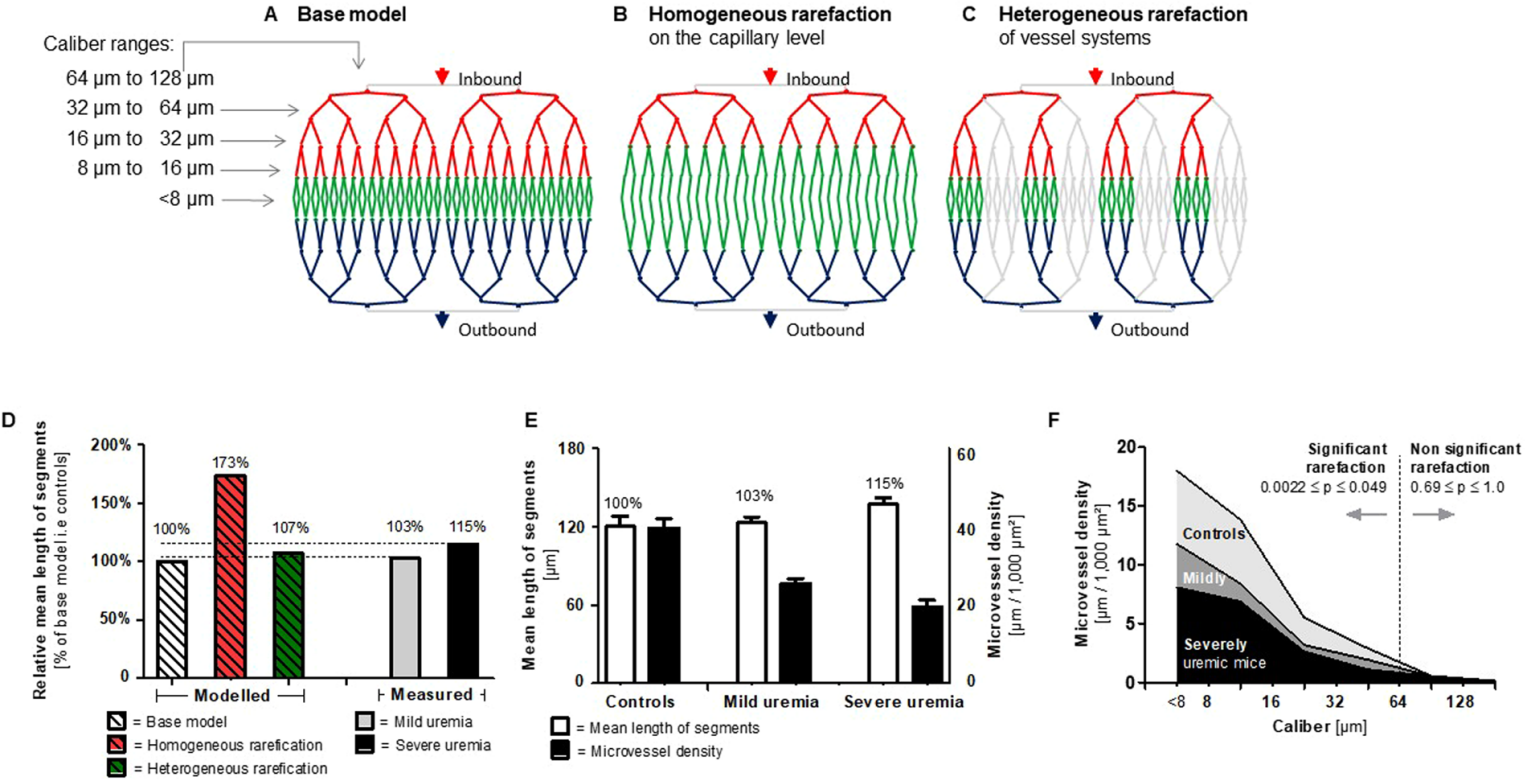
CKD-induced rarefaction is characterized by loss of coherent networks of vessel calibers <64 µm. (**A**) Model with two vessel trees, entailing arterial vessels (red), capillaries (green), and venous vessels (blue). A tree consists of nine vessel hierarchy levels with defined caliber ranges (<8 µm, 8 to 16 µm, 16 to 32 µm, 32 to 64 µm, 64 to 128 µm, and >128 µm). (**B**) Model of homogeneous rarefaction by loss of 50% of capillaries. (**C**) Model of heterogeneous rarefaction by loss of four vascular trees. (**D**) In the model of homogeneous rarefaction, the average segment length of the microvessel system (the distance between two adjacent bifurcations) is increased by 73%, and in the heterogeneous rarefaction model by 7%. (**E**) Compared to controls, measured average segment lengths were not significantly increased (only by 3% in mildly uremic, and by 15% in severely uremic mice) despite substantial microvascular rarefaction, indicating loss of coherent microvessel networks. (**F**) Microvessel densities of controls, mildly and severely uremic mice, differentiated by caliber size. Significant rarefaction in the experimental groups affects predominantly microvessels with calibers <64 µm. For larger vessels (calibers >64 µm) rarefaction is not significant.

In a theoretical model, microvascular rarefaction could proceed (A) in a homogeneous pattern of regression affecting first the most abundant smallest vessels in all networks, or (B) in a heterogeneous pattern of loss of coherent vessel systems in some, but not other networks (Fig. 2A–C). To assess the heterogeneity of a microvascular network, we determined the average segment length, i.e. the average distance between two adjacent bifurcations. In model A, assuming a 50% reduction of capillaries, rarefaction would lead to a significantly increased average segment length, but preservation of coherent vessel networks. In model B, loss of some coherent vessel networks would lead to increased heterogeneity, while the average segment length (of remaining networks) would remain nearly constant.

In our experiments, the average segment length remained almost unchanged by rarefaction (controls: 116 ± 27.9 µm, mildly uremic mice 120.2 ± 22.7 µm, i.e. 3% extension) except in severely uremic mice (133.7 ± 23.9 µm, i.e. 15% extension; p = 0.01). Thus, the microvascular network of uremic mice was not predominantly affected by a homogeneous regression of capillaries (this would have led to an increase in segment length by up to 73% in the model), but rather by loss of coherent vessel systems (vascular trees) (Fig. 2D). While this conclusion is based on model assumptions, we further investigated the degree of rarefaction in the different microvascular caliber classes. All measured segments were allocated to six arbitrarily defined caliber classes: <8 µm, 8–16 µm, 16–32 µm, 32–64 µm, 64–128 µm and >128 µm (Fig. 2A). We found that caliber classes up to 64 µm were most strongly affected by rarefaction, which mirrored their relative abundance, with most of the reduction found in the smallest caliber classes. In contrast, vessels of caliber > 64 µm were affected only to a minor extent or not at all (Supplementary Table 2, Fig. 2F), again suggesting rarefaction in CKD occurred preferentially in coherent networks of small vessels.

Our *in*-*vivo* study also permitted quantification of functional rarefaction, i.e. non-perfusion of intact microvessels. Functional rarefaction was quantified in the adenine model. We did observe temporary loss of perfusion, especially in vessels with a diameter < 64 µm, for a maximum duration of approximately 10 seconds, after which reperfusion started; about 4–6% of microvessels in mildly uremic mice and 10–13% of microvessels in severely uremic mice showed this pattern. Permanent non-perfusion (for >2 min.) was only seen in a very small fraction of microvessels (1.2% in mildly uremic and 3.9% in severely uremic mice). These observations suggest that functional rarefaction has only very little importance for the observed loss in microvascular density in CKD.

### Microvascular rarefaction results in increased diffusion distances impairing oxygen supply

**Figure 3:**
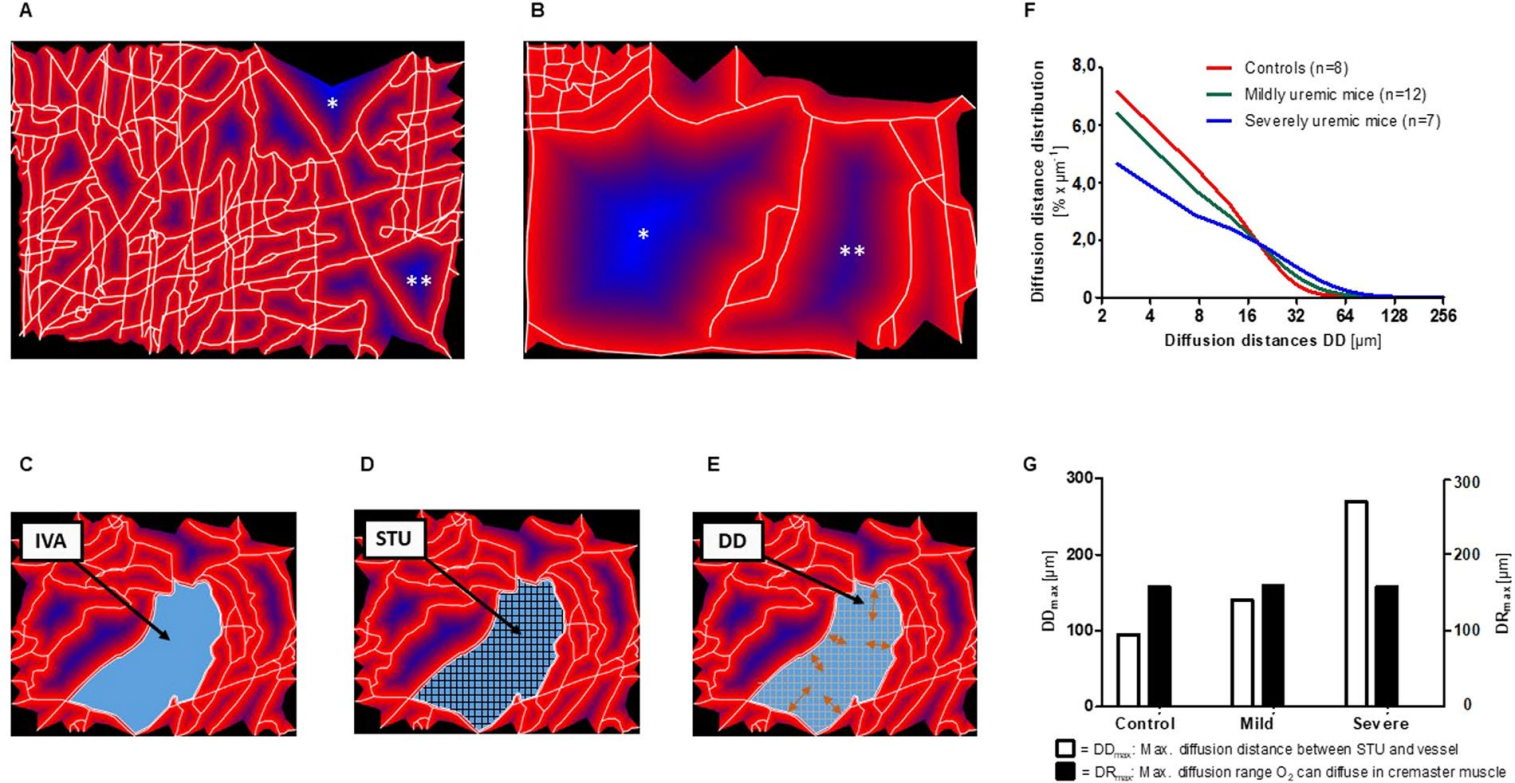
Microvascular rarefaction results in increased diffusion distances impairing oxygen supply. (**A**,**B**) Digital microvessel reconstruction (white lines). Intervascular areas (IVA) are coded in red in perivascular regions, and in blue in more distant regions; decreased color intensity indicates extended diffusion distances. (**A**) Reconstructed microvascular network of a control mouse (serum urea 46 mg/dL, microvascular density 54 µm/1,000 µm^2^). The largest IVA (*) has an area of 38,000 µm^2^, the second largest IVA (**) of 26,000 µm^2^. (**B**) Microvascular network of a severely uremic mouse (serum urea 450 mg/dL, microvascular density 12 µm/1,000 µm^2^). The largest IVA (*) has an area of 314,000 µm^2^, the second largest IVA (**) has an area of 222,000 µm^2^. (**C**) An intervascular area (IVA) is a projection surface of the muscle tissue, enclosed by reconstructed vessels. (**D**) A smallest tissue unit (STU) is the least digitally resolvable plot of an IVA (i.e. one-pixel size of 0.3 µm × 0.3 µm). (**E**) The diffusion distance DD is the distance between an STU and its closest vessel contact point. (**F**) DD distribution curves for the three experimental groups. The area under the curve corresponds to the percentage share of vessel lengths of this particular caliber range (e.g. 8 to 16 µm). At higher levels of uremia, DDs shift from shorter to longer distances. (**G**) DD_max_ is the longest DD measured in an experimental group. DR_max_ is the calculated maximal oxygen diffusion distance in the cremaster tissue of a BALB/c mouse. For severely uremic mice, DD_max_ exceeds DR_max_ indicating the existence of absolute hypoxic areas.

Since diffusion is critical for oxygen uptake in peripheral tissues, we constructed a two-dimensional digital model of the microvascular network and measured the size of intervascular areas (IVA), the diffusion distances (DD) within these IVA and their distribution (Fig. 3A–E). The size of the IVA in control animals (n = 8) was 4,004 ± 925 µm^2^, and the average DD was 11.0 ± 1.8 µm. In mildly uremic mice, the size of the IVA was 10,542 ± 3,166 µm^2^ (p = 0.0003 vs. control), and the average DD was 14.8 ± 2.1 µm (p = 0.005 vs. control). In severely uremic mice, the size of the IVA was 17,869 ± 5,356 µm^2^ (p = 0.0006 vs. control), and the average DD was 23.0 ± 3.0 µm (p = 0.002 vs. control). Thus, microvascular rarefaction in CKD mice was associated with a significant increase in IVA and DD.

The DD distribution for controls (n = 8), mildly (n = 12), and severely uremic mice (n = 7) is depicted in Fig. 3F. For small and medium DD (0–12.5 µm), control mice had an integrated area under the distribution curve (AUC) of 74.5%. Hence, 74.5% of all DD of control mice were 12.5 µm or less. For the same DD range, severely uremic mice had an AUC of only 49.5%, a 34% decrease compared to controls. For long DD (>62.5 µm) the AUC was 0.2% in controls and 6.2% in severely uremic mice. Thus, severely uremic mice showed a 31-fold increase in long DD > 62.5 µm.

To assess whether the extension of DD impairs the oxygen supply by diffusion in IVA, we measured the maximal diffusion distance (DDmax) and calculated the maximal diffusion range (DRmax) of oxygen in the cremaster muscle tissue (Fig. 3D–G). The DDmax in the experimental groups was 95 µm in controls, 140 µm in mildly uremic and 270 µm in severely uremic mice, respectively. An estimate for DRmax can be derived from the parameters diffusivity, solubility, partial pressure and consumption rate of oxygen^12^, which varies in different tissues^13^. The DRmax was calculated as 157 µm, 159 µm and 157 µm, respectively, for these groups (for calculations, see Supplementary Methods). These results compare to maximal oxygen diffusion ranges in tissue of 20 up to 200 µm, which have been calculated elsewhere using these equations^12^. Thus, DDmax values were well within the calculated DRmax in controls and mildly uremic mice, but exceeded DRmax by far in severely uremic mice (Fig. 3G). In this group, 4.3% of DD exceeded DRmax, indicating that this percentage of tissue was out of reach for oxygen supply by diffusion.

### Blood flow velocity and leukocyte rolling velocity in CKD

Blood flow velocity (V_B_) was measured in microvessels of different calibers, according to caliber ranges described above (Fig. 2A). V_B_ was significantly decreased in the uremic mice only in vessels of calibers >16 µm (Supplementary Figure 3A). For calibers between 16 and 32 µm, V_B_ of severely uremic mice was 34% below the corresponding V_B_ of controls (p = 0.027). The respective difference in V_B_ was 35% for calibers 32 to 64 µm (p = 0.024), and 36% for calibers 64 to 128 µm (p = 0.001). In vessels of calibers <16 µm, V_B_ was not significantly different between severely uremic mice and controls (p = 0.71).

Leukocyte rolling is considered the primary interaction of white blood cells with the vascular endothelium. It is known that the velocity of leukocyte rolling increases with blood flow velocity and thus with increasing vessel diameter. In non-uremic mice (controls of adenine model and sham operated), leukocyte rolling velocities were measured between 15.6 and 120.1 µm/s, and in severely uremic mice between 2.4 and 75.5 µm/s, and correlated with vessel diameter in both groups (non-uremic mice: r = 0.76; p < 0.0001; severely uremic: r = 0.63, p < 0.0002). Except for vessels with calibers 32 to 64 µm, leukocyte rolling velocity was not significantly different between non-uremic and severely uremic mice (Supplementary Figure 3B).

### CKD diminishes convective oxygen transport

The oxygen uptake rate from the microcirculation into the tissue is driven by three factors: (1) the blood flow velocity in microvessels, (2) the hemoglobin concentration (hematocrit), and (3) the arteriovenous difference in oxygen saturation of hemoglobin (avDO_2_). Eventually, the relative change in oxygen uptake is a linear function of the individual variations of these factors. To estimate impairment of oxygen uptake under uremic conditions, we measured the uremia-driven change of each factor in defined arterial and venous vessels, and determined their partial impact

**Figure 4:**
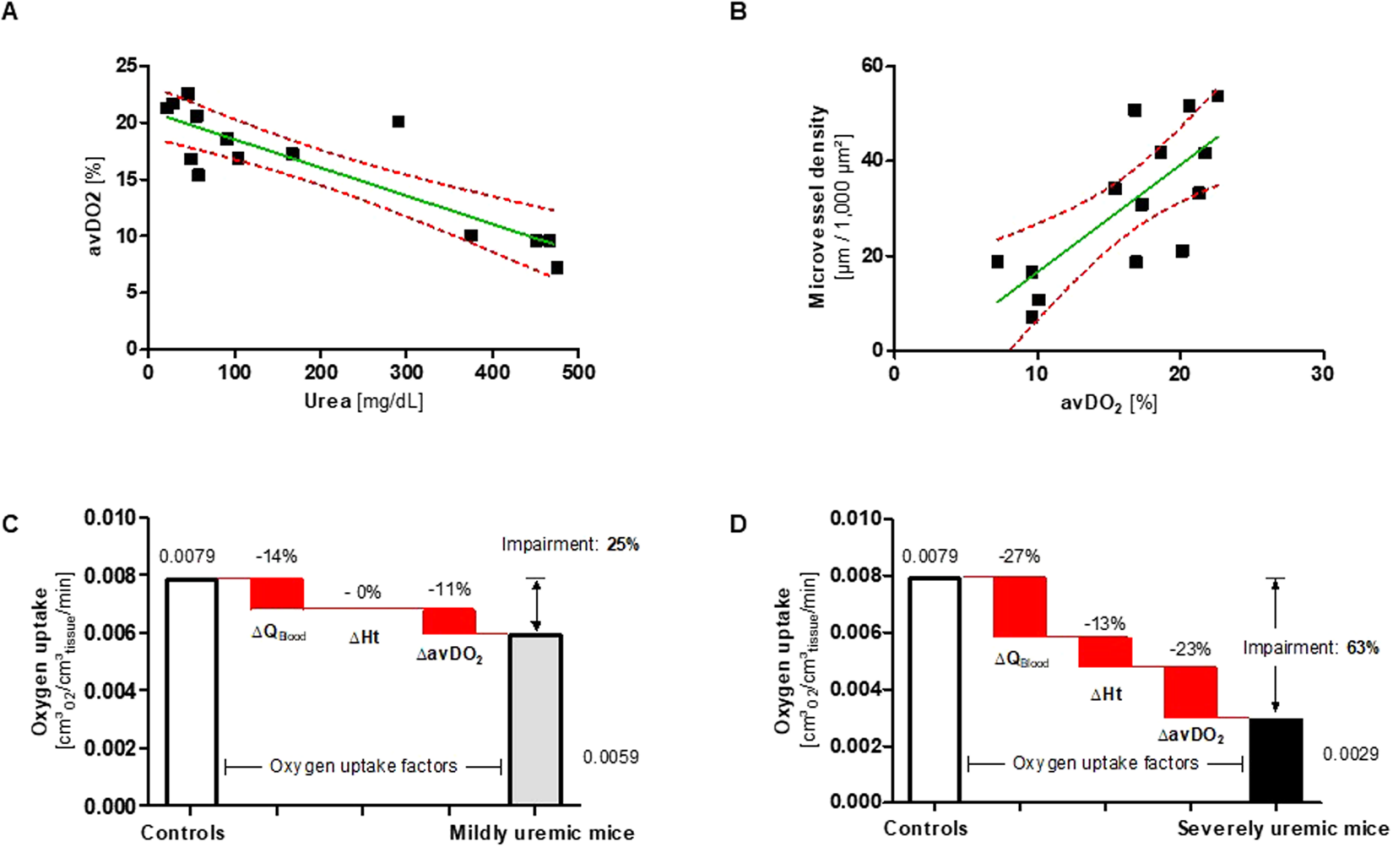
Oxygen uptake from the microcirculation is substantially impaired in CKD. (**A**) Arteriovenous difference in the oxygen saturation of hemoglobin (avDO_2_) between arterial and venous microvessels of diameters 64 µm to 128 µm was measured in controls (n = 3), mice with mild uremia (n = 2), severe uremia (n = 5), sham operated mice (n = 3), and SNX (n = 1). AvDO_2_ correlates negatively with serum urea (p < 0.0001; r = −0.87). (**B**) Microvessel density positively correlates with avDO_2_ (p = 0.0022; r = 0.75). (**C**) Oxygen uptake rate (O_2_ transition from the microcirculation into the tissue) of controls (n = 8) compared to mildly uremic mice (n = 12). The oxygen uptake rate is impaired by 25%. Three factors contribute to the impairment (assuming additive effects): (1) reduced blood flow velocity (ΔV_Blood_), (2) decreased hematocrit (ΔHt), (3) minor avDO_2_ (ΔavDO_2_). (**D**) Oxygen uptake rate of controls (n = 8) compared to severely uremic mice (n = 7), the oxygen uptake rate is impaired by 63%. Assuming additive effects, the percentage contributions of ΔVBlood, ΔHt and ΔavDO_2_ were calculated in a mass balance model based on measurements in the 64–128 µm caliber vessels (red bars) for mildly uremic (**C**) and severely uremic mice (**D**).

Blood flow velocity (Supplementary Figure 3A) and the hematocrit (Table 1) were measured as described above. The avDO_2_ between arterial and venous vessel was measured in 14 mice (3 adenine controls, 2 mice with mild uremia, 5 with severe uremia, 3 sham operated mice, 1 SNX). In total, 274 measuring points were included in the analysis. The avDO_2_ of the 64 to 128 µm caliber class was negatively correlated with serum urea levels (r = −0.87, p < 0.0001; Fig. 4A) and positively with microvessel density (r = 0.75; p = 0.002; Fig. 4B).

Importantly, variations in factors influencing oxygen uptake depend on the microvessel caliber.

Since microvessels of calibers between 64 and 128 µm comprise the largest microvessels in the cremaster muscle (Fig. 2A–C), and microvascular rarefaction is not significant on this level (Fig. 2F), an oxygen uptake impairment measured on this top level is a reasonable surrogate for the oxygen uptake impairment of the overall cremaster muscle. The factor variation on top level resulted in total oxygen uptake impairments of 25% (mild uremic mice versus controls), and 63% (severe uremic mice versus controls). Applying a physiological oxygen uptake rate of a rat cremaster muscle of 0.0079 cm³O_2_/ cm³/min to the model^14^ amounted to an oxygen consumption rate of 0.0059 and 0.0029 cm³O_2_/ cm³/min, respectively. Assuming additive effects, the relative impact of the individual factors in a mass balance model was as follows: In mildly uremic mice, 14 percentage points (out of the total 25% impairment) could be attributed to diminished blood flow and 11 percentage points to the diminished avDO_2_. Hematocrit had no impact on oxygen uptake impairment under mild uremic conditions since its variation was not significant in this group. Similarly, in severely uremic mice, 27 percentage points (out of the total 63% impairment) were attributed to reduced blood flow, 13 percentage points to the lower hematocrit, and 23 percentage points to a decreased avDO_2_ (Fig. 4C and D).

### CKD is associated with diminished vascular tone

Changes of the arterial diameter are dependent on resting vascular tone. The increase in vascular diameter was measured in 15 animals (4 controls, 5 mildly uremic and 6 severely uremic) after administering acetylcholine, adenosine, papaverine, and sodium nitroprusside. In control mice, the relative arterial diameter increase was 15.8 ± 1.5%. Mildly uremic mice showed a diameter increase of 13.2 ± 2.5% (p = 0.33 vs. controls), and severely uremic mice a diameter increase of 8.7 ± 3.6% (p = 0.02 vs. controls). Compared to controls, the change in diameter was reduced by 17% in mice with mild uremia and by a 45% in severely uremic animals. The change in vessel diameter correlated inversely with the serum urea levels (r = −0.76; p = 0.001).

### Microvascular rarefaction in the myocardium parallels rarefaction in the cremaster muscle

**Figure 5:**
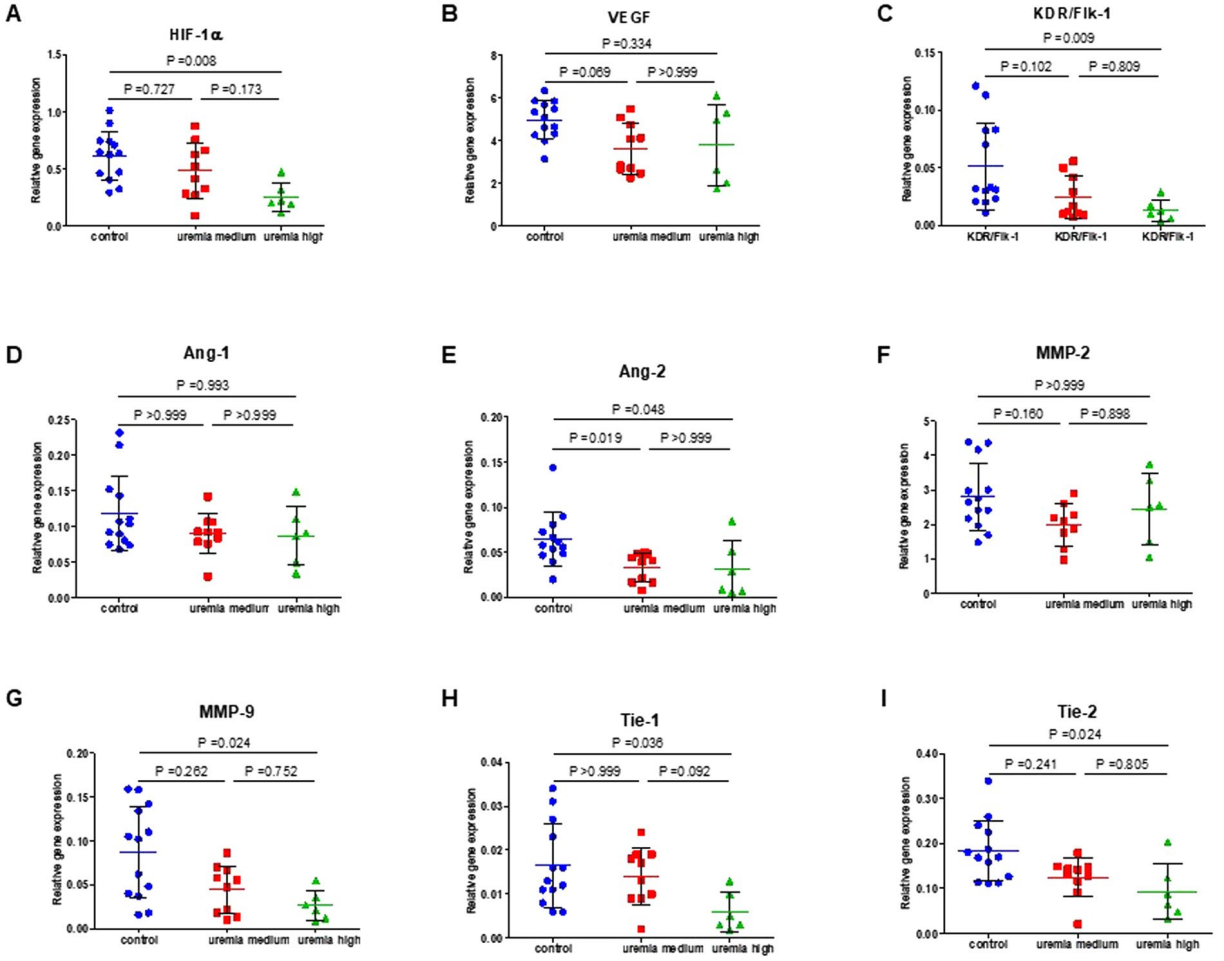
Microvessel density of uremic mice in the myocardium declines in parallel with the density in the cremaster muscle. Myocardial tissue sections were stained with an monoclonal antibody against mouse CD31 and microvascular density was expressed as percentage of positively stained area. (**A**) Microvessel density (mean + SD) in the myocardium of controls (n = 8), mildly (n = 3; *p = 0.012); and severely uremic mice (n = 6; **Mann Whitney test: p = 0.0007). (**B**) Significant inverse linear correlation of microvascular density in myocardium with urea (r = −0.73; p < 0.0022). (**C**) Close correlation of microvascular density (CD31 staining) in myocardium with microvascular density in the cremaster muscle measured by intravital imaging (r = 0.90; p < 0.0001) in identical animals. Dotted lines in (**B**) and (**C**) indicate confidence intervals.

Assuming a generalized microangiopathy induced by CKD, microvascular density was analyzed in the myocardium of mice by an independent observer in a blinded fashion (Supplementary Figure 4). The microvascular density expressed as percentage of CD31-positive myocardial tissue area of mice with mild uremia (n = 3) and severe uremia (n = 6) was significantly decreased compared to controls (n = 8), corresponding to a reduction by 50.3% and 76%, respectively (Fig. 5A). Microvessel density in the myocardium was significantly (r = −0.73; p = 0.0022) associated with blood urea levels (Fig. 5B) and microvascular density in the M. cremaster (r = 0.90; p < 0.0001) of identical animals, suggesting parallel development of CKD-associated vessel rarefaction (Fig. 5C). We could not find any evidence for apoptosis or autophagy in myocardial tissue of uremic mice or controls (Supplementary Figure 5).

### CKD induces dysfunctional angiogenesis

**Figure 6:**
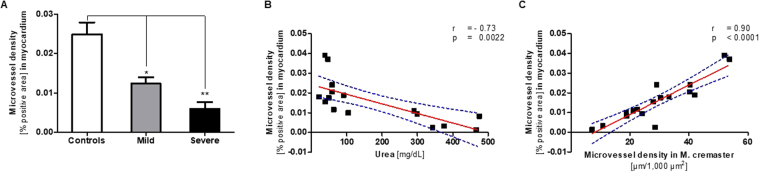
CKD suppresses transcription levels of angiogenesis regulating genes. RNA was isolated from cryoconserved heart tissues from mice (adenine-induced uremia) with mild (n = 10) or severe uremia (n = 6) and controls (n = 13) and gene transcription levels analyzed by RT-PCR and analyzed by Kruskall-Wallis test with Dunn’s test for multiple comparisons post hoc. Shown are individual values (mean ± SD).

To further study mechanisms of microvascular changes in uremia, heart tissue from mice with mild (n = 10) or severe uremia (n = 6) and controls (n = 13) was analyzed by RT-PCR for transcription levels of genes regulating angiogenesis: the transcription factor HIF-1α, the vascular endothelial growth factor VEGF-A and its receptor (KDR/Flk-1), the vascular growth factors angiopoietin 1 and 2 (Angpt-1, Angpt-2), their receptors TIE1 and TIE-2, and the matrix metalloproteinases 2 and 9 (MMP-2, MMP-9). We found a significant reduction in transcription levels of HIF-1α, Angpt-2, TIE-1 and TIE-2, Flkt-1 and MMP-9. The decrease in transcription levels of all of these genes and of the Angpt-1/Anpt-2 ratio was significantly and inversely associated with blood urea levels (HIF-1α: p = 0.0021, r = −0.55) and positively with microvascular density in the cremaster muscle (HIF-1α: p = 0.0037, r = 0.52). Transcription levels of VEGF-1, Angpt-1 and MMP-2 were not significantly different from controls (Fig. 6).

### Microvascular rarefaction in mice precedes macrovascular pathology

On cross sections of the aorta of controls and severely uremic mice, the vessel wall layers (intima, media, internal and external elastic laminae, adventitia layer) could clearly be distinguished. Two tissue sections were evaluated for each animal (n = 19, 11 uremic, 8 controls). All animals, including severely uremic mice, showed normal morphology and no calcium deposits by von Kossa staining (Supplementary Figure S4). The intima-media thickness of mice aortas was not significantly different in uremic mice and controls (48.1 ± 7.4 vs. 43.6 ± 7.1 µm; p = 0.10).

## Discussion

In this *in*-*vivo* microscopy study, we found that in an incremental manner, CKD was associated with a loss of coherent networks of microvessels resulting in a highly heterogeneous pattern of focal microvascular rarefaction, and with diminished blood flow velocity, decreased vascular tone, and impaired oxygen uptake. Furthermore, we found corresponding levels of microvascular rarefaction in the myocardium and evidence for a dysregulated angiogenesis. Altogether, these findings indicate the presence of a systemic uremic microangiopathy and chronic tissue hypoxia.

### Uremia models

Experimental uremia was induced in two well established murine models of CKD, subtotal nephrectomy and adenine feeding^15^. These models differ considerably in symptoms and severity of uremia, which in mice is best reflected by serum urea levels; serum creatinine levels tend to increase later after renal injury in mice and are much less elevated, especially in the adenine model, which results in loss of body mass^16,17^. Following subtotal nephrectomy, mice have stable decreased renal function and do not develop progressive CKD and are not hypertensive for up to 16 weeks^18,19^. In contrast, destruction of renal tissue by adenine feeding produces progressive CKD, systemic inflammation and weight loss in a time-dependent manner^16,20^. Importantly, there was a large amount of biological variation in the degree of uremia produced by both experimental protocols, permitting the investigation of the effects of CKD of different severity. Microvascular density was strongly correlated with renal dysfunction over the whole range of urea levels, independent of the experimental model and other CKD-associated conditions such as anemia (hematocrit), weight loss, and inflammation (IL-6 levels). These data indicate that the level of renal dysfunction is the main driving force for the extent of microvascular rarefaction in both experimental models. However, blood pressure increased in both experimental models and was significantly elevated in mice with severe uremia due to adenine feeding. Therefore, although blood pressure was not a significant predictor of microvascular density in the multivariate analysis, it cannot be ruled out that increases in systemic blood pressure played a role in the development of microvascular rarefaction.

### Distinct pattern of rarefaction

CKD induced a heterogeneous pattern of rarefaction, which shows similarities to previous studies by Hansen-Smith *et al*. in hypertensive rats^21^; these authors described loss of microvessel integrity due to dissociation of endothelial and smooth muscle cells in small arterioles, occurring in focal areas of the microcirculation.

We found a significant reduction in total vessel length, whereas the average segment length remained almost unchanged. In both experimental models, CKD promoted microvascular rarefaction by loss of whole networks of coherent microvessel systems, not only including capillaries (caliber 8–16 µm) but also small arterioles and venules with caliber classes up to 64 µm. Of note, recent Microfil perfusion studies of the kidney vasculature in three different murine models of progressive kidney injury showed that, independent of the model, kidney injury was associated with a progressive reduction of renal capillaries and small arteries, up to the level of the AA interlobulares, but not of larger caliber arterial vessels^22^. The interlobular arteries have a diameter range of 30–90 µm in rats^23^. These data and our study suggest that CKD promotes the loss of coherent vessel systems distal to the level of smaller arterioles, i.e. with a cutoff level about 60 µm. It is conceivable that this process originates in the supplying arteriole as suggested by the work of Hansen-Smith *et al*.^21^.

### Diffusion distances and convective oxygen transport

Uremic mice had a significant increase in intravascular areas resulting in increased diffusion distances. Severely uremic mice had maximal diffusion distances exceeding the calculated maximal diffusion range for oxygen, indicating chronic hypoxic conditions for parts of the muscle tissue. It is conceivable that chronic hypoxia, by recruiting inflammatory cells and promoting tissue fibrosis^24^ drives a vicious circle resulting in further hypoxia and organ damage^25^. Thus, rarefaction could spread in a similar way in areas of hypoxia, and this mechanism could provide a plausible explanation for the observed large avascular areas.

However, microvascular rarefaction not only impaired oxygen transport by diffusion, but also convective oxygen transport resulting in a reduced oxygen uptake rate. The avDO_2_ correlated negatively with the degree of microvascular rarefaction, indicating that lack of capillary surface area leads to a reduced unload of oxygen and thus, to a converging avDO_2_ of arterioles and venules, i.e. functional, microvascular shunting^26^. Similarly, spontaneously hypertensive rats develop arteriovenous shunts that gradually substitute normal microvascular networks^27^. Microvascular shunting in CKD could therefore aggravate and perpetuate chronic tissue hypoxia.

Massive loss of microvessels results in an increased total peripheral resistance and deceleration of the blood flow, particularly at the level of arterioles. Consequently, we observed a reduced blood flow velocity in mice with CKD, in spite of the presence of anemia, which normally accelerates blood flow.

### Impaired oxygen uptake

We calculated an oxygen uptake impairment by 25% and 63% in mildly and severely uremic mice, respectively, for microvessels with a diameter of 64–128 µm, mediated by reduced blood flow (due to rarefaction), a lower hematocrit (due to renal anemia), and a diminished avDO_2_ (due to rarefaction and functional shunting). While these calculations are based on mathematical modeling (and not direct measurements of oxygen uptake) and may not be simply translatable to humans, our observations are in line with several studies in dialysis patients showing abnormally low peak oxygen uptake, even after normalization of hemoglobin levels by erythropoietin therapy; this was explained by impaired oxygen transfer to muscle tissue mitochondria^28–30^. Our study suggests that chronic hypoxic conditions may be present in peripheral tissues even under resting conditions, depending on the severity of uremia.

### Vascular tone

Endothelium-dependent vasodilatation is impaired in uremic animals^31^ and in CKD patients^32,33^. We here show, that likewise, endothelium-independent maximal vasodilatation is significantly reduced in uremic mice and that the measured loss of vascular tone is closely associated with the severity of uremia. According to Hagen-Poiseuille’s law, this deprives the affected tissue of its ability to effectively regulate perfusion. At least three different mechanisms could explain reduced vasodilatation in CKD: (A) a reduced bioavailability of NO could be a major contributor to altered vascular responsiveness^34^. However, the use of SNP, which delivers an excess of NO, could not normalize vascular tone, suggesting other or additional mechanisms. (B) Vascular remodeling could reduce vascular compliance; the maximum achievable vessel diameter would then be reduced. While this hypothesis requires further studies of microvascular composition in CKD, it should be mentioned that skin biopsies of dialysis patients showed thickening of the basement membrane and chronic inflammatory cell infiltration in subdermal capillaries^35^. (C) Finally, microvessels in uremic tissue could be permanently dilated (and therefore have a blunted response to vasodilators) in order to compensate for prevailing hypoxia by increased blood flow. This adaptive response is well described in acute and chronic hypoxia^36^.

### Microvascular rarefaction in the heart

Depending on serum urea levels, microvessel density in cardiac and cremaster muscle of identical animals declined in a parallel, strongly suggesting a systemic “toxic” effect of uremia on the microcirculation. Although differences in local vascular density should be acknowledged^37^, a systemic effect implies a massive loss of functioning endothelium, with severe consequences for organ perfusion and metabolic homeostasis. In the heart, microvascular dysfunction could explain myocardial stunning observed in patients after volume depletion during hemodialysis^38^. While the heart is particularly vulnerable, microvascular dysfunction could be a common pathway for the well-known progressive end-organ damage in uremic patients, and this link is supported by several clinical studies documenting microvascular disease and hypoxic damage to the cardiovascular, pulmonary, cerebrovascular, and musculoskeletal system as well as cardiac and overall survival of dialysis patients^39–41^.

### Dysfunctional angiogenesis

A balance of angiogenesis-regulating genes is critical for maintenance of microcirculatory structure and function. We found a decrease in transcription levels of not only HIF-1α, the master regulator of transcriptional responses to hypoxia, but also of its target genes Angpt-2, TIE-1 and TIE-2, Flkt-1 and MMP-9, thus demonstrating lack of a core response to hypoxia. While VEGF transcription was not significantly changed in CKD mice, it should be regarded as too low for the documented amount of hypoxia. However, Flkt-1 transcription levels were significantly decreased. Downregulation of the VEGF receptor Flkt-1 has been similarly observed in coronary artery endothelial cells under hypoxic conditions^42^, indicating impaired angiogenic responses to VEGF in chronic hypoxia. While Ang-1 levels were unchanged, Ang-2 levels decreased significantly in uremic mice. Ang-1 is usually considered pro-angiogenic, Ang-2 destabilizing and anti-angiogenic, and the Ang-1/Ang-2 ratio is thought to determine a pro-angiogenic or anti-angiogenic environment. This study shows an increased (pro-angiogenic) Ang-1/Ang-2 ratio in uremic animals, and similar observations were made in omental biopsies obtained from children with CKD^11^. However, the expression of the Tie-2 receptor, which binds both angiopoietins was significantly decreased in uremic mice, indicating disruption of angiopoietin signaling with increased blood urea levels. Transcription of Tie-1, a co-regulator of Tie-2 function^43^, was also downregulated. Taken together, our study shows an incremental suppression of pro-angiogenic signaling via both, the VEGF axis and the Angpt-1-TIE-2 axis; this amounts to a reverse relationship between angiogenesis and hypoxia in CKD.

### Hypoxia, inflammation and fibrosis

Although upregulation of HIF-1α is protective in acute kidney injury^44^, long-term hypoxia-driven overexpression is associated with increased kidney fibrosis^45,46^, suggesting that downregulation of HIF-1α might be protective^47,48^. Prolonged hypoxia suppresses HIF-1α expression by several mechanisms^25^; therefore one explanation for dysregulation of angiogenesis in uremic mice could be an adaptive response to hypoxia. In addition, prolonged hypoxia may induce endothelial to mesenchymal transition, a pathway leading to vascular dropout and fibrosis in the kidney^49^ and other organs^50^ as well as CKD induced cardiac fibrosis^51^. Finally, persistent inflammation has long been identified as a major risk factor for vascular disease in CKD^52^. Both, uremia and hypoxia, enhance the expression of a multitude of pro-inflammatory mediators, further contributing to oxidative stress and endothelial damage^24^. Taken together, these data indicate profound lack of compensatory mechanisms for repair of microvascular rarefaction, as part of a vicious circle of hypoxia-driven dysregulation of angiogenesis and further stimulation of inflammatory and fibrotic mechanisms.

Our study suggests that capillary networks could be lost by CKD-induced absence of growth-supporting angiogenic signals. Deficient angiogenesis could therefore be both, a cause and a consequence of microvascular rarefaction in CKD. However, microvascular rarefaction is likely induced and perpetuated by many other mechanisms, e.g. circulating antiangiogenic factors and dysfunctional or deficient endothelial progenitor cells^53–55^. Moreover, in cultured endothelial cells, uremic serum or increased inorganic phosphate may induce apoptosis, and carbamylated low-density lipoprotein autophagy, respectively, suggesting dose-dependent toxic effects of plasma components on the endothelium in CKD^51,56,57^. *In vivo*, endothelial damage could be promoted by a plethora of uremic toxins or urea itself ^58^. Thus, published evidence suggests an active CKD-induced endothelial damage and/or regression of microcirculatory networks, but we could neither find evidence for apoptosis nor autophagy in the myocardium. However, vascular rarefaction starts within days after establishing experimental CKD^22^; apoptosis, autophagy or other mechanisms inducing vascular regression^59^ therefore may be operative early in the disease and not detectable after weeks or months as in our experimental models. In fact, recent studies in mice have demonstrated uniform rarefaction and functional alterations in peritubular capillaries within days in three experimental models of CKD^60^.

### Microangiopathy precedes macroangiopathy in mice with CKD

We show profound microvascular rarefaction in uremic mice in the absence of atheroma or calcifications in aortic cross-sections. Although there are important species differences in atherosclerosis susceptibility, limiting translation of these findings to humans, it is noteworthy that endothelial dysfunction is an early event in CKD and tissue biopsy studies have demonstrated microvascular rarefaction even in children with pre-dialysis CKD^11^. Our findings are in line with most previous studies of the microcirculation in CKD. Capillary rarefaction was documented in animals with experimental CKD in the myocardium and skeletal muscle^37,61–63^, and in CKD patients by nailfold microscopy^33^ and in the myocardium post mortem^64^ and in the kidney, where rarefaction of the peritubular capillaries is a typical feature of chronic progressive renal disease^22,60^ and chronic kidney allograft failure^65^.

Our study has several limitations. Some experiments were only performed in adenine fed mice and controls and results might not apply for other animal models or humans. Urea levels were very high in adenine fed mice, exceeding the degree of uremia observed in most humans, and thus further limiting comparisons. Oxygen uptake was not measured directly, and the degree of hypoxia was inferred from calculations of vascular network models, which may not resemble physiological structures. It is a further limitation of our study that we did not perform additional morphological or functional analyses of skeletal muscle tissue. Microvascular rarefaction has been recognized to develop in parallel with uremic myopathy^37,63^, and it seems quite possible that fatigue, muscular inactivity, fiber atrophy, metabolic abnormalities and many other factors^66^ may further contribute to decreased oxygen supply, tissue fibrosis and microvascular rarefaction in muscle, resulting in a vicious circle. Finally, although the results of our study suggest the presence of systemic microvascular disease, quantitative differences in microvascular supply between tissues cannot be ruled out.

In conclusion, our study indicates a systemic, early, and incremental effect of CKD on the microvascular architecture and function. Microvascular rarefaction, tissue hypoxia, and dysfunctional angiogenesis are key components of a vicious circle amplifying the significant impairment of vital functions of the microcirculation. These data define microvascular rarefaction and dysfunction as important therapeutic targets for future interventional studies in CKD patients.

## Methods

### Animals

*In*-*vivo* studies were performed on the microvascular network of the cremaster muscle of BALB/c mice. A total of 39 six week old male BALB/c mice with a weight of 17 to 25 g were obtained for Charles River GmbH (Sulzfeld, Germany). The animals were kept in facilities with stable environmental conditions and a 12 hour artificial light day and night cycle.

Experimental uremia was induced by either adenine feeding or subtotal nephrectomy (SNX). In the adenine model, 19 mice were fed an ROD 18 diet (LASvendi, Soest, Germany) including 0.2% adenine additive (Sigma-Aldrich, St. Lois, USA) for four weeks. Eight control mice received ROD 18 diet without additives. SNX was performed via a two stage surgical procedure. Seven mice underwent a total nephrectomy of the left kidney, 5 control animals were sham operated. Thirty minutes prior to surgery, animals received preoperative analgesia (metamizol, 200 mg/kg p.o.). For surgery, they were anaesthetized with isoflurane (4%/L O2 for induction of anesthesia, and 1.5% to 2.0%//L O2 for maintenance of anesthesia). The right kidney was removed via a dorsal incision. In sham-operated animals, the respective kidneys were exposed and shifted to achieve comparable operative stress. On recovery, tramadol (10 mg/kg s.c) was given to cover the post‐operative period. Animals were observed until full recovery from anesthesia and then returned to a clean cage. For three days mice received tramadol (2.5 mg/100 ml p.o.) via drinking water, and were monitored closely. Body weight and health status were evaluated daily. After a recovery period of two weeks, mice were subjected to a 2/3 nephrectomy of the left kidney, with analgesia and anesthesia being the same as mentioned above. After surgery, the animals were kept under standardized housing conditions for a period of 4 months. Body weight was monitored on a daily basis.

All animal experiments were approved by the Institutional Animal Care and Use Committee and the local state office of occupational health and technical safety (Landesamt für Gesundheit und Soziales, Berlin, reference G 0396/10) and carried out according to institutional guidelines.

### Cremaster Muscle Preparation

For intravital microscopy purposes i.e. cremaster muscle preparation, mice were anesthetized with urethan i.p. (1,500 mg/kg), and ketamine i.m. (50 mg/kg). During surgery or intravital microscopy, anesthesia was continuously monitored upon pedal withdrawal reflex. Appropriately, ketamine was replenished i.p. (half dose of initial anesthetic). Eventually for euthanasia purposes, pentobarbital i.p. (250 mg/kg) was administered.

All animals underwent tracheotomy to prevent airway obstruction, catheterization of the right jugular vein for infusion of 0.9% NaCl to maintain fluid balance as well as catheterization of the left carotid artery for continuous monitoring of MAP and heart rate. The scrotum was cut, the cremaster muscle exposed and fascia and connective tissue were cautiously removed. The muscle was mounted on the acrylic glass stage to a surface of about 1 cm² and covered with transparent foil (Saran, SC Johnson, Racine, USA) to minimize evaporation. The muscle tissue was regularly moistened using 37 °C tempered 0.9% NaCl solution.

### Intravital Microscopy

The cremaster muscle’s microvasculature was visualized on a modified intravital microscope (Axiotech vario, Carl Zeiss, Jena, Germany) applying a saline immersion objective (20×/0.50 W) at transmitted light, and a 1.6× microscope optical path amplification. In observation lasting several minutes, vessels, blood streams, and for small vessels, the movement of individual erythrocytes or leukocytes could clearly be visualized. A one minute sequence of vessels and blood streams was recorded on videotape using a CCD (charge coupled device) Kappa Camera system (Kappa Optronics GmbH, Gleichen, Germany) connected to a VHS recorder (DVCAM 64 PDV-64ME, Sony, Japan).

An initial visual field of 350 × 250 μm muscle tissue area was recorded at the first major bifurcation of the cremasteric artery following by further visual fields resulting in a 3 × 3 matrix area of approximately 1,000 × 750 μm (neighboring visual fields overlap of about 10 μm). In total, up to 5 cremaster areas per animal were recorded, corresponding to up to 45 visual fields. Distances between distinct cremaster areas were 1,720 μm horizontally and 2,020 μm vertically.

### Analysis of Microvascular Morphology

From the VHS recorder, the analogue film was visualized on a monitor. Applying the Software Vision 3D, the cremaster vessel areas were manually reproduced into digital network pattern files. Microcirculatory networks were defined as microvessels with diameters below 300 µm including arterioles, capillaries, and venules. In a standardized coordinate system starting point and end point of the vessel distances, lengths, diameters, points of bifurcation, and directions of the blood stream were registered. Segment length was defined as the distance between two bifurcations. The data file was edited and statistically processed in Microsoft editor Version 5.1, Micosoft Excel 2010, Matlab 2008 (MathWorks, Natick, USA), and Sigmaplot 12.0 (Systat Software, Inc., San Jose, USA) for statistical analysis. The microvascular density was defined as accumulated length of microvessels [μm] per cremaster muscle area [μm²].

### Oxygen Delivery

To estimate changes in oxygen delivery, diffusion distances (DD) within the microvascular network were calculated from a two-dimensional model constructed from a total of 999 visual fields observed in 19 animals. A vessel-enclosed area (intervascular area, IVA) in this model thus represented an avascular region of the cremaster muscle, where oxygen transport is purely by diffusion. Each IVA of the digitalized microvascular network was subdivided into pixels (size of each pixel: 0.3 × 0.3 μm^2^). To calculate a DD distribution, the distance of each IVA pixel to the nearest microvessel contact point was measured. In addition, a color-coding algorithm was applied for visualization of DD ranges.

### Blood Flow Velocity

The center line velocity of the blood flow of vessels (arteries and veins) with diameters from 12 to 110 μm was determined based on the technology of spatial correlation^67^. An asynchronous flash illumination system (model 11360-1, Chadwick-Helmuth, El Monte, USA) was used to illuminate one half-frame immediately before a CCD camera executed recordings. The next flash followed after a short delay of 5.0 ms applied for low blood velocities, or 1.1 ms applied for high blood velocities^67^.

Each vessel was recorded for a period of approximately 10 seconds during constant asynchronous flash illumination. Velocities were calculated from sequences of pairs of line intensity patterns and their spatial shifts between the lines. Blood stream peaks or variances due to heart pulsatility were numerically averaged and the center line velocity was converted into a mean velocity of blood flow (V_B_)^68^.

### Leukocyte Rolling Velocity

Rolling distances of leukocytes (per time of recording) were measured by the Vision 3D software based on recorded blood flow sequences.

### Oxygen Saturation Imaging Spectroscopy

Oxygen saturation in cremaster muscle microvessels (diameter range 64–128 µm) and hemoglobin levels were assessed by an imaging spectroscopy approach as described elsewhere^69^, taking advantage of changes in the hemoglobin absorption spectrum according to its oxygen saturation^70^. This approach allows the automatic discrimination of microvessels from tissue background. The oxygen saturation for individual microvessels can be determined in transillumination intravital microscopy. Spectral analysis was performed using a monochromator / digital camera system (TILL Photonics, Gräfeling, Germany) with a software package (TILL Vision, TILL Photonics, Gräfeling, Germany). For the calculation of oxygen saturation, the SOAP software was used^69^. Optical densities for a wavelength of 500–600 nm were analyzed. Background intensities were subtracted and the concentration of hemoglobin (Hb) and oxyhemoglobin (HbO_2_) calculated after calibration with appropriate reference absorption spectra. Spectroscopic measurement of arterial and venous oxygen saturation was performed in order to determine the avDO_2_ (arterial – venous difference in oxygen saturation).

### Vascular Tone

To achieve a complete vascular smooth muscle relaxation, a mixture of endothelial-dependent (acetylcholine) and endothelial-independent vasodilatory compounds (adenosine, papaverine, sodium nitroprusside) was applied to the cremaster muscle preparation^71^. Images of the arteries were recorded from identical positions using a digital X/Y positioning microscopy table (Elesta Elektrotechnik AG, Switzerland) before and after administering the vasodilatory cocktail. To calculate the arterial tone, the ratio of the difference between arterial diameter of maximal relaxation (D_max_) and arterial diameter at resting state (D) divided by D_max_ was used^72^.

### Blood and Tissue Sample Handling

After intravital microscopy analysis, blood samples were drawn for further analyses and heart and aorta were harvested. Organs were immediately frozen in liquid nitrogen and stored at −80 °C, or preserved in phosphate-buffered formaldehyde solution 4% (Roth GmbH, Karlsruhe, Germany) for subsequent paraffin-embedding. Paraffin blocks were sliced in 4 or 8 µm layers with a microtome and used for hematoxylin-eosin or von Kossa staining. The intima-media thickness (IMT) of mice aortas was measured using representative von Kossa stained paraffin embedded sections. Using Image J, the distance from the lumen/intima edge to the media/adventitia edge of the arteries was measured at 12 different spots per section. The average mean of these segments was taken as mean IMT ± SD.

### Quantification of microvascular density in the myocardium

Tissue imaging was performed with a digital microscope (Keyence BZ-9000) using the Analyzer Software®-II (Keyence, Neu-Isenburg, Germany). Vessels with the morphological characteristics of arterioles or venules were excluded from the analysis. After correction for brightness, thinness, and optical contrast, microphotographs were taken (1/350 sec., 10-fold magnification). Up to 72 images were then used for a high-resolution analysis of each tissue section.

Immunohistochemistry was performed on formalin-fixed tissue sections according to standard methods. Dewaxed and rehydrated tissue sections were incubated in 3% hydrogen peroxide to block endogenous peroxidases. The heat-induced antigen retrieval was performed in a pressure cooker, using the Dako REAL Target Retrieval Buffer (Dako Cytomation, Glostrup, Denmark). A monoclonal antibody against mouse CD31 (1:100) (PECAM-1) (D8V9E) XP® Rabbit mAb was purchased from Cell Signaling, Beverly, MA, USA. Bound antibody was visualized with the Vector®NovaRED™ detection kit (Vector Laboratories, Burlingame, CA, USA). Cell nuclei were counterstained with hematoxylin, and eosin and antibody diluents without primary antibodies were used as respective negative controls.

Apoptosis was measured with a polyclonal antibody against mouse cleaved caspase-3 1:300 (Asp175; Rabbit mAb, Cell Signaling Technology) and by TUNEL staining (APOBrdU Immunohistochemistry Kit, Bio-Rad Laboratories, Munich, Germany). Bound antibody was visualized with the Dako detection kits (polyclonal goat anti-rabbit immunoglobulin/AP 1:40, Dako D0487) and liquid permanent red Dako K0640. Autophagy was measured with a monoclonal antibody against mouse LC3A/B 1:1000 (D3U4C) XP® Rabbit mAb, (Cell Signaling). Bound antibody was visualized with the Dako detection kits (polyclonal goat anti-rabbit immunoglobulin/HRP 1:100, Dako P0448) and Vector®NovaRED™ (Vector Laboratories).

### Analysis of gene expressions in the myocardium

Gene expressions in murine heart tissue were determined by quantitative real-time reverse transcription-polymerase chain reaction (RT-PCR). Cryoconserved heart tissues were minced in Trizol (Invitrogen) and RNA was isolated according to the manufacturer’s protocol, treated with DNAse to remove genomic contaminations, and further purified using RNeasy columns (Qiagen, Hilden, Germany). RNA transcription, RT-PCR and calculations were performed as described^73^. The primer sequences (BioTez Berlin-Buch GmbH, Berlin, Germany) are given in Supplementary Table 3.

### Statistics

Numerical values were documented as mean ± SD or mean ± SEM. IBM SPSS version 23 and GraphPad Prism 5.01 software was used for statistical calculations. Differences between groups were tested by the Mann-Whitney test or Kruskall-Wallis test with Dunn’s multiple comparison test as post hoc. A p-value < 0.05 was considered significant. Univariate correlations were calculated by Spearman’s rank correlation coefficient. Multiple linear regression with stepwise variable selection was applied to identify factors independently associated with microvascular density.

### Data availability statement

The datasets generated during and/or analysed during the current study are available from the corresponding author on reasonable request.

## Electronic supplementary material

Supplementary Data
