# Supplementary material for: Chronic kidney disease induces a systemic microangiopathy, tissue hypoxia and dysfunctional angiogenesis
Source: Sci Rep. 2018 Mar 28;8:5317. doi: 10.1038/s41598-018-23663-1 (PMC5871820; doi:10.1038/s41598-018-23663-1)
Supplement: Supplementary file 1 — Supplementary Data [file 41598_2018_23663_MOESM1_ESM.doc]

**Supplementary Methods**

**Chronic kidney disease induces a systemic microangiopathy, tissue hypoxia and dysfunctional angiogenesis**

Hans-Ulrich Prommer, Johannes Maurer, Karoline von Websky, Christian Freise, Kerstin Sommer, Hamoud Nasser, Rudi Samapati, Bettina Reglin, Pedro Guimarães, Axel Radlach Pries, and Uwe Querfeld

Calculation of oxygen diffusion range

Under physiological conditions, vascular networks provide adequate diffusive and convective gas and solute transport. Diffusion of oxygen is only effective over short distances due to its limited water solubility. The solubility coefficient α is approximately 3
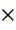
10^-5^ cm³ O₂/ (cm³ tissue x mmHg) ^1^.

An estimate of the maximal diffusion range DR_max_ [cm] is given by the equation ^2^

1.
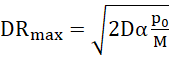


where D is the diffusion coefficient [cm²/min], α the solubility coefficient [cm³ O₂/ (cm³ tissue x mmHg)], p₀ the partial pressure of oxygen [mmHg] at the start of the diffusion route and M the oxygen consumption rate [cm³ O₂/(cm³ × min)].

The following parameters were gathered from the literature for the calculation of DR_max_ in the murine cremaster muscle: Dα = 6,0 * 10^-10^ [cm³ O₂/(cm x **s** x mmHg)] ^3,4^; M = 0.0079 [cm³ O₂/(cm³ × min)] ^5^. The partial pressure of oxygen p₀ in the capillary was calculated from the hemoglobin saturation in arterial and venous vessels (diameter 64 to 128 µm) by applying the oxygen-hemoglobin dissociation curve. For controls, mildly and severely uremic mice, the calculated p₀ was 27.0 mmHg, 19.3 mmHg und 10.7 mmHg, respectively. Using these parameters, the DRmax was calculated as 157 µm, 169 µm and 158 µm, respectively.

**Modeling of oxygen uptake**

In commonly applied mathematical models, microvascular networks are designed either as tree-like or network-like vessel structures. However, physiologically, they resemble irregular complex structures ^1^. For the computation of oxygen uptake in tissues, we employed a tree-like pattern (**Fig. 2A**).

The convective transport of oxygen in a blood stream (
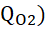
 is the product of the convective blood flow
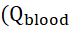
) and the oxygen content in the blood
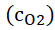
.

1.
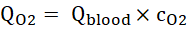


The oxygen content of blood comprises both, oxygen bound to hemoglobin (Hb) as well as oxygen dissolved in serum. We ignored the latter because of its insignificant quantity. The amount of oxygen bound to Hb is a product of the oxygen saturation of Hb (
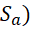
, the Hb concentration, and the oxygen binding capacity of Hb expressed by the Huefner coefficient.

1.
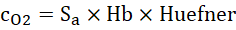


Hence, for steady state conditions, convective oxygen transport in a vessel with an inner diameter D and a mean blood flow velocity
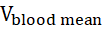
 can be expressed as:

1.
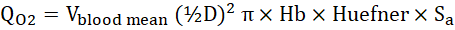


The mean blood flow velocity of
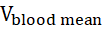
 can be derived from the blood flow velocity, measured at the center line of the vessel
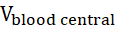
 applying an approximation equation dependent on hematocrit (Hct), and vessel diameter D [µm] ^6^:

(5)
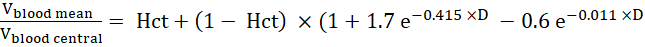
)

Oxygen uptake U_O2 uptake_ of a tree-like model is the amount of oxygen penetrating from the convective blood flow into the surrounding tissue between point A and point B.

The oxygen uptake in this model can be described in an equation as

(6)
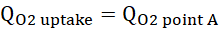
-
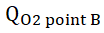


Assuming identical diameters, blood flow velocities, and Hct at point A and point B, and a difference in Hct oxygen saturation between point A and B expressed by avDO_2_ (arterial-venous difference in O_2_ saturation), the oxygen uptake rate in this model depends primarily on three variable factors: (1) mean blood flow velocity, (2) hemoglobin, and (3) the arterial-venous difference in O_2_ saturation.

(7)
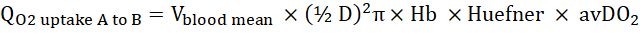


Assuming a constant MCH (mean corpuscular hemoglobin), the hemoglobin is proportional to hematocrit (Hct). Hence, applying the measured data to the model allowed a calculation of the impairment of oxygen uptake of mildly and severely uremic mice compared to controls.

1 Pries, A. R. & Secomb, T. W. Making microvascular networks work: angiogenesis, remodeling, and pruning. *Physiology (Bethesda, Md.)* **29**, 446-455, doi:10.1152/physiol.00012.2014 (2014).

2 Secomb, T. W., Hsu, R., Dewhirst, M. W., Klitzman, B. & Gross, J. F. Analysis of oxygen transport to tumor tissue by microvascular networks. *International journal of radiation oncology, biology, physics* **25**, 481-489 (1993).

3 Pries, A. R., Reglin, B. & Secomb, T. W. Structural adaptation of microvascular networks: functional roles of adaptive responses. *Am J Physiol Heart Circ Physiol* **281**, H1015-1025 (2001).

4 Bentley, T. B., Meng, H. & Pittman, R. N. Temperature dependence of oxygen diffusion and consumption in mammalian striated muscle. *Am J Physiol* **264**, H1825-1830 (1993).

5 Johnson, P. C., Vandegriff, K., Tsai, A. G. & Intaglietta, M. Effect of acute hypoxia on microcirculatory and tissue oxygen levels in rat cremaster muscle. *J Appl Physiol (1985)* **98**, 1177-1184, doi:10.1152/japplphysiol.00591.2004 (2005).

6 Pries, A. R. & Secomb, T. W. Microvascular blood viscosity in vivo and the endothelial surface layer. *Am J Physiol Heart Circ Physiol* **289**, H2657-2664, doi:10.1152/ajpheart.00297.2005 (2005).

**Supplementary Table 2**

**Microvascular rarefaction differentiated by caliber**

**
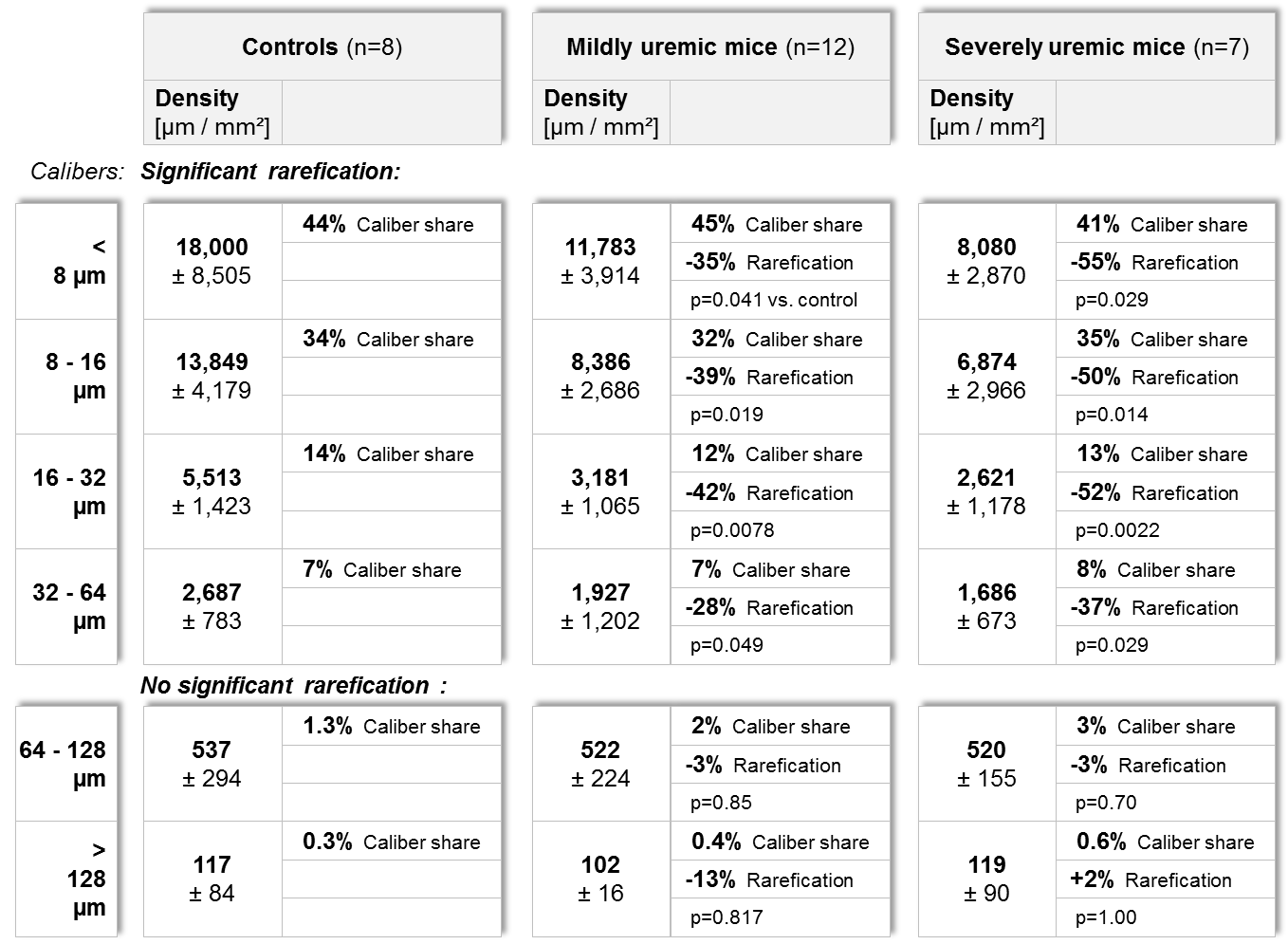
**

**Supplementary Table 3**

Primer sequences

| Gene | forward primer | reverse primer |
| --- | --- | --- |
| HIF-1α | 5'-TCATCAGTTGCCACTTCCCCAC-3' | 5'-CCGTCATCTGTTAGCACCATCAC-3' |
| VEGF | 5'-ACTTGTGTTGGGAGGAGGATGTC-3' | 5'-AATGGGTTTGTCGTGTTTCTGG-3' |
| KDR/Flk-1 | 5'-TTCCCCCCTGGAAATCCT-3' | 5'-ACAGACCCGGCCAAACAA-3 |
| Angpt-1 | 5'-AACCTCACCCTGCAAAGATG-3' | 5'-CACAGATGGCCTTGATGTTG-3' |
| Angpt-2 | 5'-CAAGGCACTGAGAGACAC-3' | 5'-TGCGCTTCAGTCTGGTACAC-3' |
| MMP-2 | 5'-AAAGGACTCGGGTTGTCTGA-3' | 5'-ACTTGGTTCTCCTCCATCCA-3' |
| MMP-9 | 5'-TCCCGAGAGTCCAACTCACT-3' | 5'-CATCTCACCTGGAGGACACA-3' |
| TIE-1 | 5'-GTGCCACCATTTTGACACTG-3' | 5'-CAGGCACAGCAGGTTGTAGA-3' |
| TIE-2 | 5'-GATTTTGGATTGTCCCGAGGTCAAG-3' | 5'-CACCAATATCTGGGCAAATGATGG-3' |

**Supplementary Figures**

Supplementary Figure 1

**Supplementary Figure 1: Serum creatinine levels in experimental groups.** Bars indicate mean + SEM in controls (n=8), mice with mild (n=12) and severe uremia (n=7) due to adenine feeding, sham-operated (n=5) or 5/6 nephrectomized (n=7) animals.

Supplementary Figure 2


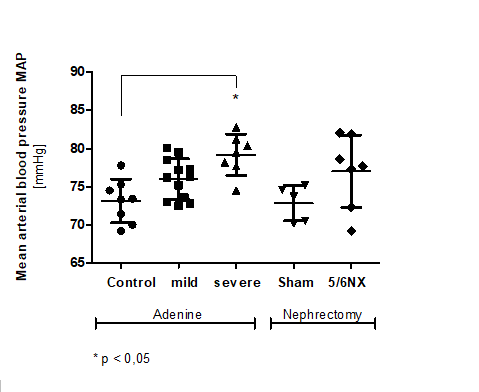


**Supplementary Figure 2: Mean arterial blood pressure (MAP) in experimental groups.** Bars indicate mean + SD in controls (n=8), mice with mild (n=12) and severe uremia (n=7) due to adenine feeding, sham-operated (n=5) or 5/6 nephrectomized (n=7) animals. MAP was measured by an A. carotis catheter after preparation of the M. cremaster (1-2 h) and before intravital microscopy. During the 2 to 3 hour procedure of intravital microscopy, MAP dropped continuously by about 10 to 15 mmHg, but differences between groups remained unchanged (data not shown).

Supplementary Figure 3

**Supplementary Figure 3: Blood flow velocity and leukocyte rolling velocity.** (**A**) Blood flow in microvessels of defined caliber ranges (mean±SEM) of controls (n=4) and severely uremic mice (n=5). Blood flow velocity in larger uremic vessels (caliber >16 µm) is significantly decreased compared to controls (p=0.027 for calibers 16 µm to 32 µm; p=0.024 for calibers 32 µm to 64 µm; p=0.001 for calibers 64 to 128 µm). In smaller vessels (caliber <16 µm), blood flow velocity in the uremic animals is not different compared to controls (0.71 ≤ p ≤ 0.78). (**B**) Leucocyte rolling velocity. With exception of caliber class 32 µm to 64 µm (p<0.0001), leukocyte rolling velocity (controls versus severely uremic mice) is not significantly different (Mann Whitney test: p=0.45 for calibers 8 µm to 16 µm, p=0.14 for calibers 16 µm to 32 µm, and p=0.086 for calibers 64 µm to 128 µm).

Supplementary Figure 4


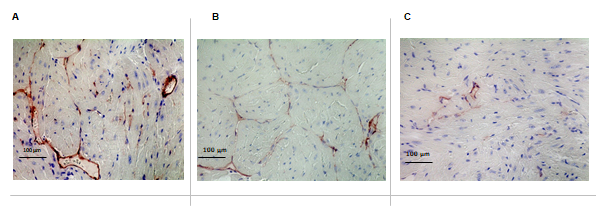


**Supplementary Fig 4: CKD induces microvascular rarefaction in the myocardium.** Myocardial tissue sections were stained with an monoclonal antibody against mouse CD31 (dark red), and microvascular density was expressed as percentage of positively stained area**.** The microvascular density of mice with mild uremia (n=3) and severe uremia (n=6) was significantly decreased compared to controls (n=8) **(A). s**ham-op, 40-fold magnification, Scale Bar 100 µm (**B)** mildly uremic mouse, 40-fold magnification, scale bar 100 µm (**C)** severely uremic mouse, 40-fold magnification, scale bar 100 µm.

Supplementary Figure 5


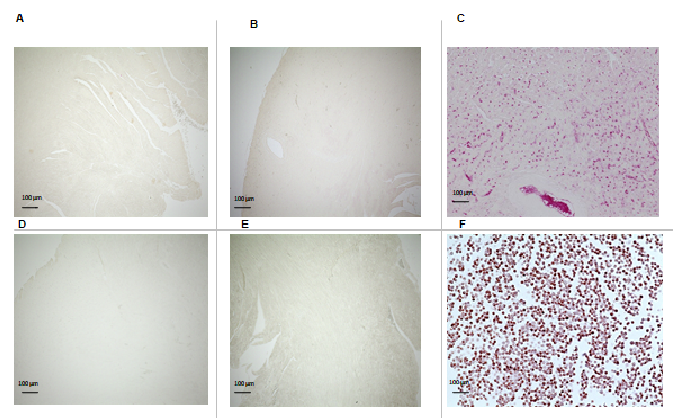


**Supplementary Figure 5: : No evidence for apoptosis or autophagy in the myocardium of mice with CKD**. Myocardial tissue sections were stained with an with a polyclonal antibody against mouse cleaved caspase-3 for the detection of apoptosis (A-C) and with a monoclonal antibody against mouse LC3A/B for the detection of autophagy (D-F). (A) mildly uremic mouse, 20-fold magnification, scale bar 100 µm (B) severely uremic mouse, 20-fold magnification, scale bar 100 µm (C) positive control, rat myocardial tissue (5/6 nephrectomy, high-dose calcitriol treatment) (D) mildly uremic mouse, 20-fold magnification, scale bar 100 µm (E) severely uremic mouse, 20-fold magnification, scale bar 100 µm (F) IHC – positive staining with LC3A/B in HeLa cells treated with 50 μM Chloroquine (positive control).

Supplementary Figure 6

**Supplementary Figure 6: No evidence of macrovascular pathology in uremic mice**. While substantial microvascular rarefaction was evident in the myocardium and the cremaster muscle, histological cross sections of the aorta of uremic animals appear normal and show no signs of calcification. The IMT of mice aortas was not significantly different in uremic mice and controls (48.1+7.4 vs. 43.6+7.1 μm; p=0.10). (**A**) Von Kossa staining of the aorta from a control mouse. (**B**) Von Kossa staining of the aorta from a severely uremic mouse (bars = 250 μm, 10-fold magnification).
